# Atypical Anti-Glomerular Basement Membrane Disease

**DOI:** 10.1016/j.ekir.2023.03.010

**Published:** 2023-03-21

**Authors:** Joyita Bharati, Yihe Yang, Purva Sharma, Kenar D. Jhaveri

**Affiliations:** 1 Department of Nephrology, Post Graduate Institute of Medical Education and Research, Chandigarh, India; 2 Division of Kidney Diseases and Hypertension, Department of Medicine, Glomerular Center at Northwell Health, Donald and Barbara Zucker School of Medicine at Hofstra/Northwell, Great Neck, New York, USA; 3 Department of Pathology, Donald and Barbara Zucker School of Medicine at Hofstra/Northwell, Manhasset, New York, USA

**Keywords:** anti-GBM disease, atypical, IgA-mediated, IgG4-mediated, linear IgG, monotypic anti-GBM

## Abstract

Atypical anti-glomerular basement membrane (anti-GBM) disease is characterized by linear immunoglobulin G (IgG) deposition along the GBM without circulating IgG anti-GBM antibodies. Compared to classic anti-GBM disease, atypical anti-GBM disease tends to be milder with a more indolent course in certain cases. Moreover, pathologic disease pattern is much more heterogenous in atypical anti-GBM disease than in the classic type, which is uniformly characterized by diffuse crescentic and necrotizing glomerulonephritis. Although there is no single well-established target antigen in atypical anti-GBM disease, the target antigen (within the GBM) and the autoantibody type are hypothesized to be different from the classic type. Some patients have the same antigen as the Goodpasture antigen that are detected only by a highly sensitive technique (biosensor analysis). Some cases of atypical anti-GBM disease have autoantibodies of a different subclass restriction like IgG4, or of monoclonal nature. Antibodies targeting antigen/epitope structure other than the Goodpasture antigen can be detected using modified assays in some cases. Patients with IgA- and IgM-mediated anti-GBM disease are known to have negative circulating antibodies because conventional assays do not detect these classes of antibodies. A significant proportion of cases with atypical anti-GBM disease do not have any identifiable antibodies despite extensive evaluation. Nevertheless, extensive evaluation of atypical autoantibodies using modified assays and sensitive techniques should be attempted, if feasible. This review summarizes the recent literature on atypical anti-GBM disease.

Anti-GBM disease is an autoimmune disorder with the target antigen present within specific basement membranes, such as GBM and alveolar basement membrane. Anti-GBM disease is rare with an incidence of about 1 per million population per year.^1^ However, it accounts for approximately 15% of various causes of crescentic glomerulonephritides, and prompt treatment has significantly improved survival in the recent past.^1^ Presentation is abrupt and severe, with approximately 90% of patients having rapidly progressive glomerulonephritis, with or without lung hemorrhage. Strong linear Ig G deposition along the GBM on direct immunofluorescence (IF) is the pathologic hallmark and basis for the diagnosis of anti-GBM disease. Histologically, it typically shows diffuse crescents and fibrinoid necrosis. Patients with 100% crescents on kidney biopsy who require dialysis and show kidney failure at presentation have a dismal kidney survival rate of 8% at 1 year of follow-up.^2^ This makes anti-GBM disease one of the most aggressive forms of glomerulonephritides. Serum anti-GBM antibodies are detected in approximately 90% of patients. Anti-GBM disease is observed to be abrupt in onset and recurrence is uncommon. Circulating anti-GBM antibodies are pathogenic^3^ and directly associated with disease severity. Rare cases of disease recurrence, before and after a kidney transplant, that correlated with circulating anti-GBM antibodies, have been reported.^4^^,^^5^ However, reports describing severe relapse of the disease without the appearance of circulating antibodies in patients with previous positive serology are also known.^6^ In the last decade, isolated case reports and series^7^^,^^8^ describing similar IF findings as classic anti-GBM disease on kidneys but without other typical features such as aggressive clinical course and/or diffuse crescentic glomerulonephritis and/or circulating anti-GBM antibodies (tested by conventional assays) have increasingly emerged and are termed “atypical anti-GBM disease”^9^ (Table 1). A subset of patients with atypical anti-GBM disease have linear monotypic Ig deposits on IF without other features of monoclonal gammopathy of renal significance. This review compiles the recent literature on atypical anti-GBM disease to understand the disease pattern and guide patient management.

**Table 1:** Comparison of reported literature on polytypic atypical anti-GBM disease in the last decade (2012–2022)

Author/yr	N	Age (yr)	Peak serum creatinine (mg/dl)	Presenting symptoms/syndrome	Light microscopy	Class/subclass of Ig on immunofluorescence	Serology	Immunosuppressive treatment	Outcome-kidney
Troxell and Houghton^9^	4	23–64	0.75–7.1	Macrohematuria: 1 Fever: 1 Hemoptysis: 1 Constitutional: 1	Mesangial sclerosis: 1 Focal crescents: 2 Diffuse crescents: 1 IFTA: 4 Interstitial nephritis: 2	IgG dominant, subclass available in 1 showed IgG1 dominance	Negative: 1 Weakly positive: 1 Positive: 2	PLEX/CYC/steroids: 2 PLEX: 1 Steroids/Methotrexate: 1	Improved serum creatinine: 2 Normalized serum creatinine: 1 ESKD: 1
Nasr *et al.*^7^	10	18–85	2.4 (median)	Nephrotic syndrome: 5 (50%) Macrohematuria: 30%	MPGN: 3, MesPGN: 2, EPGN: 4, FSGS: 1, Focal crescent: 4, Diffuse crescent: 0, IFTA: 8, TMA: 6	IgG1 dominant:2 IgG4 dominant:3 IgG1/IgG4 codominant:1	Negative in 9/10 tested	Steroids /CYC:2 Steroids/MMF: 3 Steroids/CYC/PLEX: 1	1-year kidney survival: 67%
Fernandes *et al.*^12^	1	27	5.13	Hemoptysis, dyspnea	Diffuse necrotizing with focal crescents	IgG dominant (subclass NA)	Negative	Steroids/CYC/PLEX	Improved serum creatinine
AlSowailmi *et al.*^13^	1	27	3.13	AKI, nephrotic-range proteinuria	MPGN with focal crescents	IgG dominant (subclass NA)	Weakly positive	Steroids/CYC/PLEX	Improved serum creatinine
Liang *et al.*^10^	19	15–61	1.8	Nephrotic syndrome: 37% AKI:63%, Lung involvement: 16%	MesPGN: 6, MPGN: 2, EPGN: 2, FSGS: 3, Focal crescents: 11, Diffuse crescents: 4, IFTA: 84%	IgG4 dominant: 7 IgG1 dominant: 6 IgG4 and IgG1 codominant: 2 NA: 2	Negative	Steroids: 3 Steroids/CYC: 3 Steroids/MMF: 2 Steroids/MMF/tacrolimus: 1 Steroids/Thalidomide: 1	ESKD: 32%
Singhal *et al.*^14^	1	37	0.76	Hemoptysis and dyspnea	Focal fibrinoid necrosis, mild IFTA	IgG4 dominant	Weakly positive	Steroids/PLEX	Chest symptoms normalized
Sporinova *et al.*^15^	1	24	12.04	AKI, nephrotic-range proteinuria, hemoptysis and dyspnea	Diffuse crescentic and necrotizing MPGN	IgG4 dominant	Negative	Steroids/CYC/PLEX/RTX	ESKD (chest symptoms improved)
Adapa *et al.*^16^	1	46	10.8	Episodic macrohematuria, AKI	Diffuse heterogenous crescents and fibrinoid necrosis	IgG4 dominant	Negative	Steroids/CYC	ESKD
Shen *et al.*^8^	60	51.7 ± 15.6 (mean)	1.61	AKI: 45%, nephrotic syndrome: 31.7%, hemoptysis: 5%, Macrohematuria: 6.7%	Focal crescents: 20 Diffuse crescents: 5 MPGN: 6, MN: 8, IgAN:12, AAV: 4, FSGS: 3, TMA: 1, TBMN: 1 IFTA: 96.7%, interstitial nephritis: 91.7%	IgG1: 45.8% IgG2: 35.6% IgG4: 18.6% IgG3: 11.9%	Negative	Steroids: 53.3% Cytotoxic drugs: 30% PLEX: 6.7%	1-year kidney survival: 83.3%
Zhong *et al.*^17^	1	38	1.01	Occasional hemoptysis, microhematuria, nephrotic-range proteinuria	Cellular crescents and segmental necrosis of glomeruli	IgG (subclass NA)	Negative	Steroids/CYC/PLEX/tacrolimus	Proteinuria reduced, chest symptoms normalized
Elshirbeny *et al.*^18^	1	37	2.4	RPGN, nephrotic-range proteinuria, macrohematuria	Diffuse crescentic MPGN with mild IFTA	IgG dominant (subclass NA)	Negative	PLEX/CYC/steroids	Improved serum creatinine
Jamboti *et al.*^19^	1	30	0.7	Microscopic hematuria, hemoptysis with dyspnea	Normal	IgG dominant (subclass NA)	Negative	RTX/steroids/PLEX	Normal (+no chest symptoms)
Ramesh *et al.*^20^	1	28	1.3, 8.3 (during relapse)	Edema, Nephrotic-range proteinuria; RPGN and hemoptysis during relapse	Diffuse crescentic	IgG (subclass IgG4 was negative)	Weakly positive, negative (during relapse)	Steroids/CYC/PLEX	Improved serum creatinine (hemoptysis resolved)
Kyriazis *et al.*^11^	1	58	5.8	AKI after immune checkpoint inhibitor (nivolumab)	Focal crescentic and proliferative	IgG dominant (subclass NA)	Negative	Steroids/CYC	ESKD
Guo *et al.*^21^	1	38	4.35	RPGN	Diffuse crescentic, IFTA: 30%	IgG dominant	Negative	Steroids/PLEX	Improved serum creatinine
Tamura *et al.*^22^	1	43	1.44	Episodic macrohematuria	Focal crescents, endocapillary hypercellularity	IgG1 dominant	Negative by ELISA, positive by IIF	Steroids/CYC/PLEX	Normalized serum creatinine
Javaugue *et al.*^23^	1	73	4	AKI, nephrotic-range proteinuria after immune checkpoint inhibitor therapy (pembrolizumab)	Focal crescents, fibrinoid necrosis with endocapillary hypercellularity, moderate IFTA	IgG2 dominant	Negative	Steroids/PLEX/RTX	ESKD

AAV, anti-neutrophil cytoplasmic antibody-associated vasculitis; AKI, acute kidney injury; CYC, cyclophosphamide; EPGN, endocapillary proliferative glomerulonephritis; ESKD, end-stage kidney disease; FSGS, focal segmental glomerulosclerosis; IFTA, interstitial fibrosis tubular atrophy; IgAN, IgA nephropathy; IIF, indirect immunofluorescence; MesPGN, mesangial proliferative glomerulonephritis; MMF, mycophenolate mofetil; MN, membranous nephropathy; MPGN, membranoproliferative glomerulonephritis; PLEX, plasma exchange; RPGN, rapidly progressive glomerulonephritis; RTX, rituximab; TBMN, thin basement membrane nephropathy; TMA, thrombotic microangiopathy.

### Pathogenesis of Atypical Anti-GBM Disease

Atypical anti-GBM disease comprises 8% to 12% of all anti-GBM disease cases.^7^^,^^9^ As seen in the classic type, atypical anti-GBM disease has been described mostly in Asians and the white population, with a slight predominance in men.^7^, ^8^, ^9^, ^10^ Although a significant number of patients can have a heterogenous clinical-pathologic presentation, atypical anti-GBM disease is usually a milder and slower disease with less aggressive pathologic features setting it apart from the aggressive pattern of classic anti-GBM disease. Target antigens and autoantibodies seem to differ from those in classic anti-GBM disease in most cases.

The antigen in classic anti-GBM disease, also called Goodpasture antigen, comprises cryptic epitopes of the noncollagenous 1 (NC1) domain of the α3 subunit of type IV collagen.^24^ The cryptic epitopes, Ea and Eb, are critical amino acid residues (17–31 and 127–141, respectively) of the α3NC1 monomers. Dissociation of the alpha3 monomer from the hexamer structure results in exposure of cryptic epitopes to host immune system.^25^ Although genetic association and environmental triggers^26^ are known to be associated with classic anti-GBM disease, such associations are not yet studied systematically in patients with atypical anti-GBM disease.

Several methods for testing circulating anti-GBM antibodies include indirect immunofluorescence assay, enzyme-linked immunosorbent assay (ELISA), chemiluminescence, radioimmunoassay, multiplex bead test, western blot, and biosensor system. The sensitivity and specificity of commercial immunoassays, which measure the Goodpasture antigen, are 95% to 100% and 91% to 100%, respectively.^27^ ELISAs are more sensitive than immunofluorescence assay.^28^^,^^29^ Western blots use native GBM containing the NC1 domain of all alpha chains of type IV collagen, unlike ELISA, which uses denatured recombinant antigens comprised the NC1 domain of the α3 chain. The high diagnostic accuracy of commercial assays to detect serum anti-GBM antibodies could be limited to classic anti-GBM disease because most patients in the studies had acute nephritic syndrome and/or lung hemorrhage, preventing generalizability.^30^ About 2% to 8% of patients with anti-GBM disease have negative serology on rigorous testing. Nasr *et al.*^7^ found undetectable circulating anti-GBM antibodies on ELISA, indirect immunofluorescence, and western blot in all patients with atypical anti-GBM disease. Following changes in the antigen or autoantibody composition could result in a negative serology in atypical anti-GBM disease.

#### Composition of Epitope/Antigen

Conformational epitopes on the alpha3NC1 domain of type IV collagen, other than Ea and Eb, were detected using nonreducing western blot analysis in anti-GBM disease patients with negative serology on ELISA.^31^ Antibodies against the NC1 domain of other alpha chains like alpha1, alpha4, or alpha5 chains of type IV collagen or to the NC1 domain of alpha345 hexamers^32^ can cause rare cases of anti-GBM disease. Linear epitopes of the collagenous domain of type IV collagen or antigens other than type IV collagen, such as entactin, also cause anti-GBM disease. Could specific triggers be factors behind the change in the target antigen/epitope within the GBM? For example, immune checkpoint inhibitors ipilimumab and nivolumab are reported to be associated with atypical anti-GBM disease^24^ characterized by negative circulating anti-GBM antibodies. What makes this entity atypical, unlike the anti-GBM disease developing after anti-CD52 monoclonal antibody (alemtuzumab) use, is not yet known.^33^ Modifying the existing assays to detect epitopes and antigens beyond the Goodpasture antigen can aid the diagnosis in many such cases of atypical anti-GBM disease.

#### Affinity of Antibody

Serum anti-GBM antibodies are shown to correlate with clinical and pathologic severity of anti-GBM disease.^1^^,^^34^^,^^35^ However, some patients have high-affinity autoantibodies trapped in the kidneys and present with low titer in circulation. This low titer is not detectable in conventional assays, thereby giving false-negative results.^36^^,^^37^ High-affinity antibodies bind to the GBM strongly and dissociate slowly. Therefore, despite negative circulating autoantibodies, such patients have persistent kidney injury. Autoantibodies could also have a low affinity for the substrate in the assay. A biosensor analysis system or antigen-inhibition ELISAs can detect the affinity-binding characteristics of such antibodies, which are missed on conventional commercial assays.^38^^,^^39^

#### Type of Antibody

Nonspecific polyclonal antibodies in the plasma could alter the detection of anti-GBM antibodies.^40^ Polytypic antibodies comprising more than 1 subclass of IgG are the norm in classic anti-GBM disease. IgG1 is the most dominant IgG subclass in classic anti-GBM disease, with an equal preponderance of IgG3^41^ and IgG4.^42^, ^43^, ^44^ Although IgG1 and IgG4 were the most common IgG subclasses in atypical anti-GBM disease, dominance of one IgG subclass is common. In addition, IgG2 is reported frequently in atypical anti-GBM disease^7^, unlike classic anti-GBM disease. Both IgG2 and IgG4 are weak activators of the complement system and anti-GBM antibodies found in normal human serum also belong to IgG2/IgG4 subclass.^45^ Cases of atypical anti-GBM disease presenting as nephrotic syndrome and having focal segmental glomerulosclerosis (FSGS) on kidney biopsy had IgG2 and IgG4 subclasses, likely explaining their less aggressive course. These cases did not have any other clinico-pathologic characteristics of anti-GBM disease. Given that Ig classes other than IgG are not detected on commercial assays, serology is negative in IgA-mediated and IgM-mediated anti-GBM disease despite having clinical features like that of classic anti-GBM. Moreover, for unknown reasons, some atypical anti-GBM diseases with monotypic IgG deposits do not have detectable circulating antibodies.

### Pathology

Deposition of autoantibodies along the GBM leads to complement activation and inflammation followed by rupture of the GBM and fibrinoid necrosis in the endocapillary tuft. Leakage of proinflammatory plasma into the Bowman’s space causes parietal epithelial cell activation and crescent formation, typically diffuse involving >50% of the glomeruli.^46^ Diffuse crescentic and necrotizing glomerulonephritis with a normal appearance of glomeruli without crescents is the prototypical histopathology in classic anti-GBM disease. Bright (intensity of ≥ 2+, on a scale of 0–3+) linear Ig deposition along the GBM on IF and the absence of corresponding electron-dense deposits along the GBM on electron microscopy, are the hallmarks of the classic anti-GBM disease, except in cases of advanced glomerular injury where GBM structure is altered because of several breaks. Atypical anti-GBM disease is characterized by a histopathology picture comprising the hallmarks of classic anti-GBM disease, that is, bright linear Ig deposits along the GBM and absence of electron-dense deposits, but without the typical light microscopy findings of diffuse crescentic and necrotizing glomerulonephritis. Segmental linear staining of tubular basement membrane in atypical anti-GBM disease is often appreciated, similar to classic anti-GBM disease. The light microscopy appearance of atypical anti-GBM is heterogenous, unlike classic anti-GBM disease. Nasr *et al.*^7^ noted hypercellularity in the mesangium or endocapillary areas in all biopsies of atypical anti-GBM disease. Endocapillary proliferative glomerulonephritis (GN), mesangial proliferative GN, and membranoproliferative GN patterns were noted in 45%, 30%, and 15% of patients, respectively, of the 20 cases described.^7^ Similarly, Liang *et al.*^10^ noted mesangial proliferative GN in 63%, endocapillary proliferative GN in 10.5%, and membranoproliferative GN in 10.5% of patients, respectively. Whereas focal crescents and/or fibrinoid necrosis were noted in 40% of the cases in the series by Nasr *et al.*,^7^ none of them had diffuse crescentic necrotizing GN. Patients with nephrotic syndrome had FSGS concurring with mesangial hypercellularity and global foot process effacement on electron microscopy. Further, nodular glomerulosclerosis with occasional focal crescents and strong linear IgG along the GBM has been reported in 3 patients with history of heavy smoking.^47^ Tubular atrophy and interstitial fibrosis were noted in almost all (84%, 97%, and 100%),^7^^,^^8^^,^^10^ and chronic arterial/arteriolar injury was noted in most (75% and 98.3%) patients with atypical anti-GBM disease.^7^^,^^8^ Interstitial inflammation was frequent too (65% and 91.7%). Global glomerulosclerosis was reported in approximately 80% of patients with atypical anti-GBM disease in a retrospective study,^10^ reflecting a more chronic process than the monophasic and transitory nature of classic anti-GBM disease. In addition, glomerular thrombotic microangiopathy, characterized by subendothelial zone widening by electron-lucent material on electron microscopy, was noted in 40% of cases.^7^ However, none of these patients had glomerular or vascular thrombi. There were no systemic findings of microangiopathic hemolytic anemia or thrombocytopenia. Complement C3 staining was present (trace or 1+) in 55%^7^ and 65%^8^ of cases, respectively, reflecting complement system activation by the autoantibodies in approximately half of the patients, unlike its universal presence in classic anti-GBM disease. Patients with crescents had complement C3 staining universally, emphasizing the pathogenic role of complement activation in more severe atypical anti-GBM disease.^8^ Electron-dense deposits corresponding to Ig deposition on IF were universally absent, similar to classic anti-GBM disease.^7^ More than half (58.3%) of patients in the study from China had concurrent glomerular diseases like IgA nephropathy, FSGS, membranous nephropathy, thrombotic microangiopathy, anti-neutrophil cytoplasmic antibody-associated vasculitis, which likely explains the high prevalence (55.9%) of electron-dense deposits in respective locations in this study.^8^ Taken together, the subtle clinical features and heterogenous pathologic findings different from that of classic anti-GBM disease, along with the absence of an animal disease model, make one doubt if the linear IgG deposits are the reason behind the glomerular alterations in atypical anti-GBM disease. And does this entity truly belong to the spectrum of anti-GBM disease?^48^ Further, acquired T-cell sensitivity to the alpha 3 chain of type IV collagen, in the absence of anti-GBM antibodies, has been shown to cause crescentic GN in animals with presumed anti-GBM nephritis.^49^ This finding, if confirmed, could also be used to argue against the pathogenic role of linear Ig deposits in atypical anti-GBM disease. We believe that an added statement describing important pathologic findings associated with the linear IgG deposits, rather than only stating atypical anti-GBM disease, would best serve to identify this entity until new evidence emerges on the direct role of these deposits in the pathogenesis.

### Clinical Course

Classic anti-GBM disease presents abruptly with severe kidney with or without lung involvement. Nephritic syndrome and rapidly progressive glomerulonephritis are typical clinical manifestations of classic anti-GBM disease, characterized by a rapid clinical deterioration of the glomerular filtration rate. Patients present with rapid onset of oliguria or anuria followed by volume overload, often with hemoptysis. Atypical anti-GBM disease is a milder disease with an indolent course. Although kidney dysfunction was common (53%–75%), the median serum creatinine at presentation was mildly elevated (1.61–1.9 mg/dl)^7^^,^^8^^,^^10^ in patients with atypical anti-GBM disease. Moreover, severe kidney dysfunction requiring dialysis was infrequent (0%, 8.3%),^7^^,^^8^ unlike >two-thirds of patients with classic anti-GBM disease requiring dialysis at presentation.^48^ The prevalence of microhematuria was similar in both atypical (63.3%–95%)^7^^,^^8^^,^^10^ and classic anti-GBM disease,^50^ macroscopic hematuria occurred in a variable number (6.7%–20%) of atypical anti-GBM disease cases. Median proteinuria was higher than that in classic anti-GBM disease, with a more frequent prevalence of nephrotic-range proteinuria (42%–53%), likely implying a more chronic process causing glomerulosclerosis and more severe podocyte injury without rapidly shutting down glomerular filtration completely.^7^^,^^8^^,^^10^ Nephrotic syndrome was reported in approximately one-third of such patients, and all of them had FSGS as the dominant light microscopy finding.^7^^,^^8^^,^^10^ Hypertension was reported in 68% to 75% of the patients with atypical anti-GBM disease,^7^^,^^10^ unlike the scanty prevalence of hypertension in the classic disease. Despite a high prevalence of positive smoking history (53%–58%), lung hemorrhage was either absent^7^ or present in a few cases (5%–16%),^8^^,^^10^ unlike classic anti-GBM disease having lung involvement in 40% to 60% of patients.^1^ The reason behind the low prevalence of lung hemorrhage in atypical anti-GBM disease is not understood. In patients with atypical anti-GBM disease who had lung hemorrhage, kidney biopsy showed crescents, and kidney outcome was poor.^8^ Rarely, atypical anti-GBM disease having pulmonary manifestation and linear IgG staining of GBM without any kidney manifestation is also observed.^44^ Similarly, cases with negative serology but severe kidney injury are reported because of atypical anti-GBM disease.^39^ Therefore, although most atypical anti-GBM disease cases have milder diseases, severe kidney dysfunction and lung hemorrhage are also known in some patients. The absence of serum anti-GBM antibodies can delay diagnosis and lead to poor outcomes if a kidney biopsy is not sought early. An algorithm showing the approach to management of suspected atypical anti-GBM disease is shown in Figure 1.

**Figure 1:**
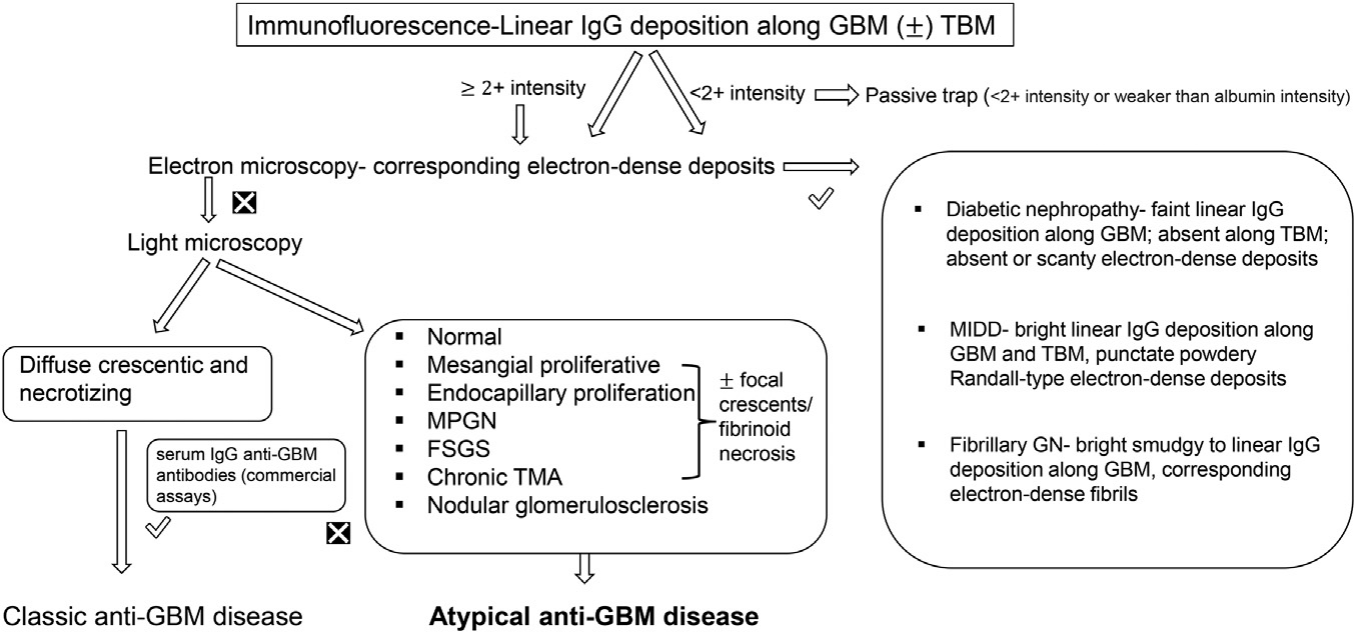
Algorithm showing the approach to management of suspected atypical anti-GBM disease. FSGS, focal segmental glomerulosclerosis; GBM, glomerular basement membrane; GN, glomerulonephritis; MIDD, monoclonal immunoglobulin deposition disease; MPGN, membranoproliferative glomerulonephritis; TBM, tubular basement membrane.

#### Treatment Options

Treatment of atypical anti-GBM disease has been heterogenous, including nonimmunosuppressive conservative therapies like renin-angiotensin-aldosterone blockers monotherapy (43.3%), immunosuppressive therapy like steroids, cyclophosphamide, mycophenolate mofetil, tacrolimus, rituximab (50%–70%), and rarely plasmapheresis (6.7%–10%).^7^^,^^8^ About one-quarter of patients progressed to end-stage kidney disease during a median follow-up of 15 to 35.7 months.^7^^,^^8^^,^^10^ The 1-year kidney survival (83.3%–85%) and patient survival (93%–95%),^7^^,^^8^ respectively, were better than classic anti-GBM disease (<10%–25%). Although short-term kidney survival appears favorable, kidney dysfunction persists in most cases. Therefore, longer follow-ups of such patients would provide practical data on prognosis.

### Various Forms of Atypical Anti-GBM Disease

#### IgA-Mediated Anti-GBM Disease

Although IgA-mediated anti-GBM disease is not a variant of atypical anti-GBM disease (because the deposited Ig in the GBM is IgA and not IgG), false-negative serology on conventional assays could initially mimic atypical anti-GBM disease. IgA-mediated anti-GBM disease is rare, with only 16 case reports in the literature to date,^51^, ^52^, ^53^ an additional case of IgG and IgA-mediated anti-GBM disease was recently reported.^54^ Further, Borza *et al.*^55^ described an elderly man with monoclonal IgA autoantibody mediated anti-GBM disease, which was associated with plasma cell dyscrasia. Clinical and pathologic manifestations are similar to classic IgG-mediated anti-GBM disease, however, circulating IgA anti-GBM antibodies are not detected on commercial assays similar to atypical IgG-mediated anti-GBM disease. Unlike the favorable 1-year kidney outcomes in treated patients with classic IgG-mediated disease, IgA-mediated anti-GBM disease was observed to have worse short-term outcomes because most patients required maintenance dialysis on follow-up despite treatment.^53^ Two groups of investigators tried to understand the specificity of the autoantigen in IgA-mediated anti-GBM disease.^52^^,^^55^ The IgA autoantibodies were found to react against cryptic linear epitopes in the collagenous domain of alpha1 and alpha2 chains of type IV collagen. This finding could explain the dominant presence of linear IgA staining along the tubular basement membrane and Bowman’s capsule. Others have reported the antigen target of IgA anti-GBM antibodies to be the NC domain of the alpha5 and alpha6 chain of type IV collagen.^56^ A delay in the diagnosis or the unmet need to target the disease differently might be factors responsible for the dismal prognosis of this variant of anti-GBM disease.

#### IgG4-Associated Anti-GBM Disease

IgG4-mediated anti-GBM disease is typically associated with favorable kidney outcomes.^57^^,^^58^ The variance of IgG4 antibodies from other IgG subclasses makes the disease pattern different. Weak classical complement pathway activation and weak binding to IgG Fc receptors on mononuclear by IgG4 makes it less likely to cause aggressive inflammatory injury.^58^ Anti-GBM disease mediated by IgG4 autoantibodies is known to be associated with milder clinical kidney dysfunction and often lacks demonstrable circulating anti-GBM antibodies by commercial assays. Modified assays that detect quaternary epitopes within alpha345 hexamers detected IgG4 subclass restricted anti-GBM antibodies in a patient with atypical anti-GBM disease.^35^ Ohlsson *et al.*^57^ described 4 cases of IgG4-mediated anti-GBM disease who presented with severe lung hemorrhage. All 4 patients were young women with either negative standard ELISA (for IgG anti-GBM antibodies), weakly positive standard ELISA, or rapidly fluctuating ELISAs that did not correlate with clinical disease severity. Specific ELISA against IgA, IgM, and IgG subclasses was undertaken promptly, which showed IgG4 anti-GBM antibodies that showed increased reactivity in the absence of a denaturing agent, confirming the reactivity of IgG4 toward the intact α345 hexamer. Kidney dysfunction was noted in 2 of the 4 patients with crescentic GN that improved with immunosuppressive therapy. Cui *et al.*^58^ described a similar case of IgG4-mediated anti-GBM disease in a young male with intermittent lung hemorrhage and mild kidney involvement in the form of microscopic hematuria and proteinuria. However, standard ELISA for anti-GBM antibodies against the α3 chain was positive (although weakly) in this patient, pointing toward the heterogenous nature of target antigens in IgG4-mediated anti-GBM disease. Although IgG4 antibodies are typically noninflammatory, the pathogenesis of lung hemorrhage and crescentic GN in some of these cases could be related to lectin complement pathway activation by IgG4 or another unknown mechanism(s). Therefore, in patients with lung hemorrhage without severe kidney involvement and doubtful circulating anti-GBM antibodies, testing for IgG4 anti-GBM antibodies by special assays should be considered.

#### Monotypic Atypical Anti-GBM Disease

Monotypic Ig deposits along the GBM, defined based on light chain restriction on IF of kidney tissue, are increasingly observed to cause a disease pattern similar to atypical anti-GBM disease. Patients typically lack circulating anti-GBM antibodies and have a milder presentation. In the study by Nasr *et al.*,^7^ monotypic deposits were defined as one type of immunoglobulin with either kappa or lambda light chain restriction. IgG1 and lambda light chain restriction was noted in two-thirds of cases of atypical anti-GBM disease with monotypic deposits.^7^ Similar isolated case reports showed monotypic deposits in patients presenting as atypical anti-GBM disease, such as 1 case with IgG3-lambda^9^ and 3 cases with IgG1-kappa^59^, ^60^, ^61^ restriction, respectively. Monoclonal IgA and IgM autoantibodies such as IgA1-kappa,^55^ IgA-lambda,^7^ and IgM-kappa^7^ are also reported. The patient with IgA1-kappa described by Borza *et al.*^55^ had a plasma cell dyscrasia and presented with mild kidney dysfunction having nonspecific light microscopic findings (without diffuse crescents), which remained stable with treatment over 4 to 5 years; however, it rapidly deteriorated to ESKD later. Of the 2 patients with IgM-kappa monotypic deposits along the GBM, 1 had plasma cell dyscrasia as evidenced by serum monoclonal protein (IgG-lambda) and bone marrow showing lambda-restricted plasmacytosis. However, the discrepancy between the type of monoclonal proteins (IgG-lambda in serum/bone marrow and IgM-kappa in kidneys) brings ambiguity in terming the IgM autoantibodies as pathogenic. In the series by Nasr *et al.*,^7^ light microscopy findings of the monotypic cases overlapped with those of polytypic cases except that crescent formation (which was universally “focal”) were seen in only 1 patient (10%) with monotypic deposits as compared to 70% of cases with polytypic deposits. The significance of this finding is unclear, because clinical outcomes and patterns did not differ between monotypic and polytypic forms of the disease. Unlike monoclonal gammopathy of renal significance, Randall-type deposits and diffuse bright linear staining of tubular basement membrane are not seen ultrastructurally in monotypic atypical anti-GBM disease.^7^ In only 1 patient, there was a concordant monoclonal protein in the serum and in the kidneys.^7^ Serum-free light chains were normal in all patients. Treatment was individualized, similar to the polytypic atypical anti-GBM disease, except that bortezomib and rituximab were more commonly used in patients with the monotypic type. The 1-year kidney and patient survival rates were similar in polytypic and monotypic diseases, respectively.^7^ Does this entity represent a form of monoclonal gammopathy of renal significance? It remains to be decided as new evidence on its pathogenesis and long-term course evolves. A summary of the differences between the classic and the atypical form of anti-GBM disease is shown in Figure 2.

**Figure 2:**
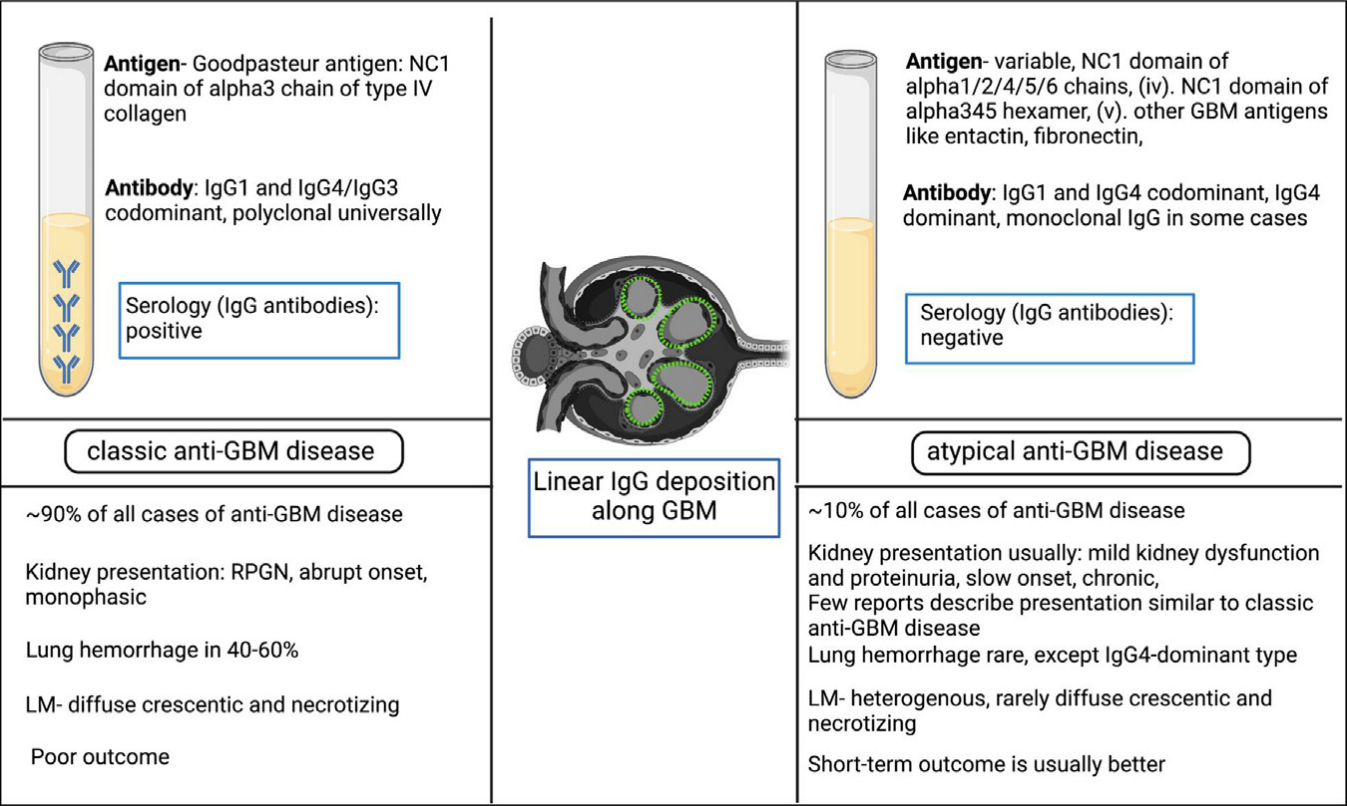
Summary of the differences between the classic and the atypical form of anti-GBM disease. GBM, glomerular basement membrane; NC1, noncollagenous 1; RPGN, rapidly progressive glomerulonephritis.

### Differential Diagnoses for Linear IgG Deposition Along the GBM

Patients with diabetic nephropathy, monoclonal immunoglobulin deposition disease (MIDD), and fibrillary GN can have linear IgG deposition along the GBM (Figure 3). A passive linear trap of IgG along the GBM because of loss of negative charge from altered GBM structure is known in diabetic kidneys. This usually appears fainter than the IgG staining in anti-GBM disease. A comparison with albumin staining can help differentiate nonspecific passively trapped IgG (which is weaker or equal to albumin staining intensity) in diabetics from anti-GBM disease and MIDD. Moreover, owing to their dominance in the serum, IgG1 is the dominant passively trapped IgG subtype in the glomeruli. Therefore, dominance of IgG2, IgG3, or IgG4 subtypes in glomeruli are unlikely to be passively trapped. Mild linear IgG trapping is described in otherwise healthy old populations. Electron microscopy would help differentiate MIDD and fibrillary GN from anti-GBM disease. Linear IgG deposits along the GBM/ tubular basement membrane in MIDD are due to the physicochemical properties of the Ig chain and not because of autoantibody response to GBM antigen in situ. Whereas established cases of MIDD are associated with punctate powdery electron-dense deposits along the basement membrane, some cases of MIDD may have very subtle or even absent immune deposits on electron microscopy despite strong IF staining^62^ resembling anti-GBM disease, so called “MIDD by IF only.”^47^ Fibrillary GN is known to present with pseudolinear IgG deposits along the GBM on IF,^63^^,^^64^ although a smudgy appearance of the immune deposits is characteristic. About a quarter of cases of diffuse crescentic fibrillary GN show linear GBM staining for IgG mimicking anti-GBM nephritis.^47^ Electron microscopy showing fibrillary electron-dense deposits along the GBM makes the diagnosis of fibrillary GN straightforward. Nonspecific linear Ig deposits should be further analyzed with elution studies of the kidney tissue to confirm the nature of Ig as anti-GBM antibodies if the initial evaluation does not point to a diagnosis.

**Figure 3:**
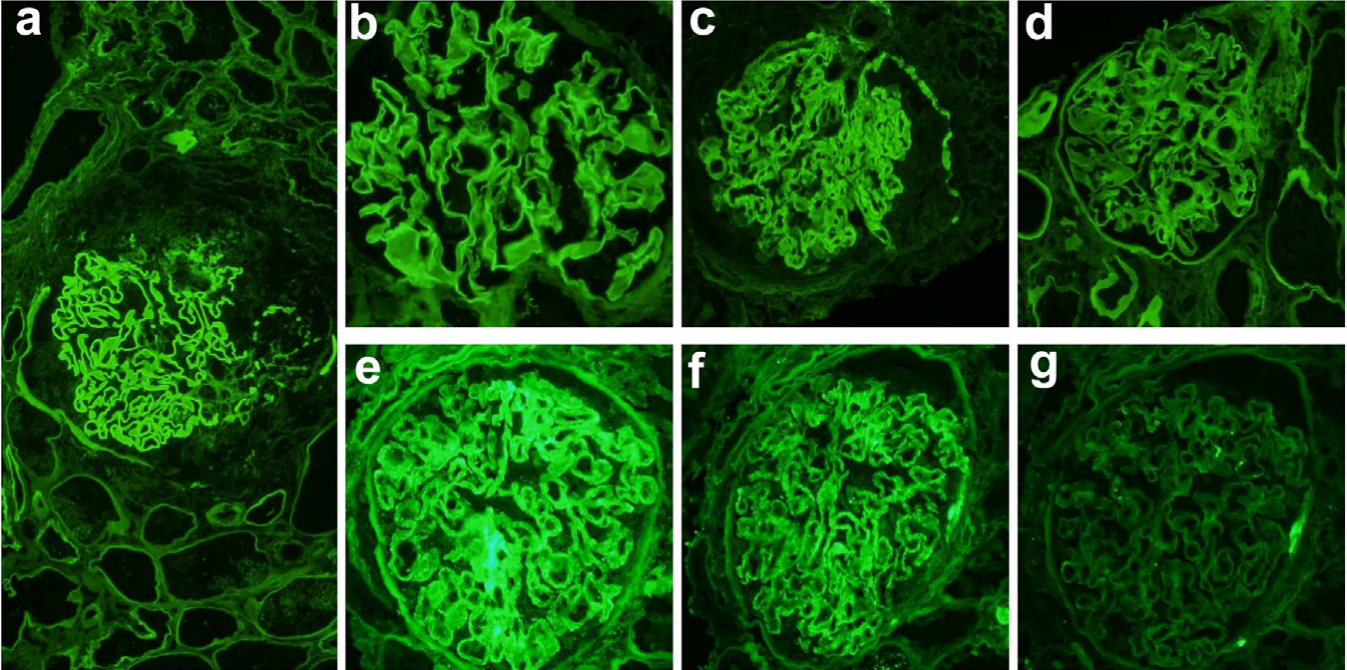
(a) Classic anti-GBM disease with strong IgG staining along GBM; (b) IgG reactivity along GBM in fibrillary glomerulopathy; (c) linear IgG staining along GBM in an atypical anti-GBM disease; (d) faint linear IgG entrapment along GBM in diabetic nephropathy; _(e–g) IgG linear stain along GBM with kappa restriction. (e) strong IgG staining along GBM; (f): strong kappa staining along GBM; (g) negative lambda stain. (magnification: 200X)

### Conclusion

Atypical anti-GBM disease is a variant of the classic anti-GBM disease characterized by linear Ig deposition along the GBM on kidney biopsy and lack of circulating antibodies. Other causes of linear Ig deposition along the GBM must be excluded based on concomitant pathology and clinical background. Kidney dysfunction is usually milder and evolves slower than classic anti-GBM disease; however, presentation as rapidly progressive glomerulonephritis has also been reported. Proteinuria and nephrotic syndrome are more frequent than classic anti-GBM disease. Light microscopy shows heterogenous patterns like mesangial and/or endocapillary proliferative GN, membranoproliferative glomerulonephritis, FSGS, mesangial sclerosis; and glomerular endothelial changes resembling thrombotic microangiopathy are common in atypical anti-GBM disease. Diffuse crescentic and necrotizing GN is uncommon. Differences in the entire target antigen or epitope structure and/or type of antibody can result in negative serology on commercial assays. Modified assays to detect a wide range of antigens or epitopes or antibodies and more sensitive techniques like biosensors can unmask circulating anti-GBM antibodies in some cases and should be attempted. The monotypic subtype of atypical anti-GBM disease needs further study to confirm if it is a form of monoclonal gammopathy of renal significance. Treatment of atypical anti-GBM disease is individualized and typically consists of immunosuppressive agents used to treat classic anti-GBM disease or monotherapy with renin-angiotensin-aldosterone blockers. A prospective multicenter study on a large cohort of patients with atypical anti-GBM disease would be worthwhile to understand its pathogenesis, clinical course, and outcome

## Disclosure

KDJ reports consultancy agreements with Secretome, George Clinicals, PMV pharmaceuticals, and Calliditas. KDJ is a founder and co-president of the American Society of Onco-Nephrology (ASON); KDJ reports honoraria from the American Society of Nephrology, and UpToDate.com; reports serving on the editorial boards of CJASN, AJKD, CKJ, Journal of Onconephrology, Kidney International, and NDT; reports serving as Editor-in-Chief of ASN Kidney News and section editor for onconephrology for Nephrology Dialysis Transplantation. Other authors have nothing to disclose.
